# Using an intelligent control scheme in the open-source design on non-invasive rehabilitation platform of physical therapy for patient

**DOI:** 10.1016/j.mex.2022.101659

**Published:** 2022-03-05

**Authors:** Ha Quang Thinh Ngo

**Affiliations:** ^1^Department of Mechatronics, Faculty of Mechanical Engineering, Ho Chi Minh City University of Technology (HCMUT), 268 Ly Thuong Kiet, District 10, Ho Chi Minh City, Viet Nam; a ^2^Vietnam National University Ho Chi Minh City (VNU-HCM), Linh Trung Ward, Thu Duc District, Ho Chi Minh City, Viet Nam

**Keywords:** Mechanical design, Open-source design, Physical therapy, Non-invasive intrumentation, Intelligent control

## Abstract

In the context of global pandemic, hospital or healthcare center is very sensitive for patients. However, the injuried patients must maintain their medical orders to rehabililate as soon as possible, otherwise, the clinical treatment could be interruptted. To overcome these troubles, an open-source design of mechanical machine for non-invasive method (NIPPT) is firstly introduced. The advantages of our approach are low-cost, available in the market and satisfy the medical requirements. The structure and motion of wrist are initially analyzed to obtain the dimensional sizes of mechanical details. Owing to computational mechanics, the architecture of hardware components is determined and manufactured. Later, sensing modules such as torque sensor, positioning sensor and limit sensor are integrated while powerful microprocessor provides the abilities of rapid calculation, small sampling time and stable operation. Then, the model-free control scheme such as fuzzy PID (F-PID) is embeded into hardware in order to drive the mechanism appropriately. Due to the simple implementation and servo lag phenomenon, this controller is expected to adapt with the uncertainties and unknown environment. Based on this design, several medical exercises are validated in the experimental tests. It is obviously seen that the proposed approach is feasible, proper and possible for clinical applications.

## Graphical abstract

**Figure:**
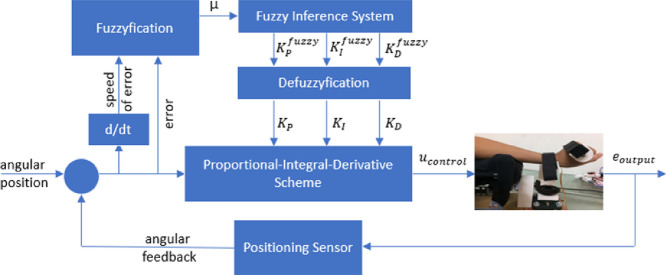

**Specifications table**

**Table:** 

**Subject Area**	Engineering
**More specific subject area**	*Bio-engineering, Mechatronics design, Motion control*
**Method name**	*The modified fuzzy control integrating PID scheme with real-time performance in medical application*
**Name and reference of original method**	Method 1: Using the recursive least square algorithm to determine the impedance parameters of human arm, the reference trajectory [1] can be generated in the case of healthy human. Then, the fuzzy logic strategy manipulate to track the desired path. *Method 2: Proposing an effective solution to take care of patients* from *far, a wrist rehabilitation system ensuring the two wrist movements, was investigated [**2**]. The fuzzy controller was employed to control the robot action according to the pain felt by the patient. From the database achieved from monitoring patients, the physiotherapists could carry out a decision from their offices.*
**Resource availability**	*Software: Solid Works, Proteus and Matlab* *Hardware: Some mechanical parts such as bearing, coupling and so on*

*Method details *[Methodological protocols should be in sufficient detail to be replicated. There is no word limit! You can include figures, tables, videos – anything that you feel will help others to reproduce the method. The main focus of the paper should be on the technical steps required for this method, more than results; where appropriate, guide the reader through the procedure and provide all extra observations or ”tricks” alongside the protocol. Results and Discussion are not sections included in the MethodsX format. However, providing data that validate the method is valuable and required. This section could become a “method validation” paragraph within the Method Details section.]*

## Mechanical analysis of proposed platform

**Fig. 1:**
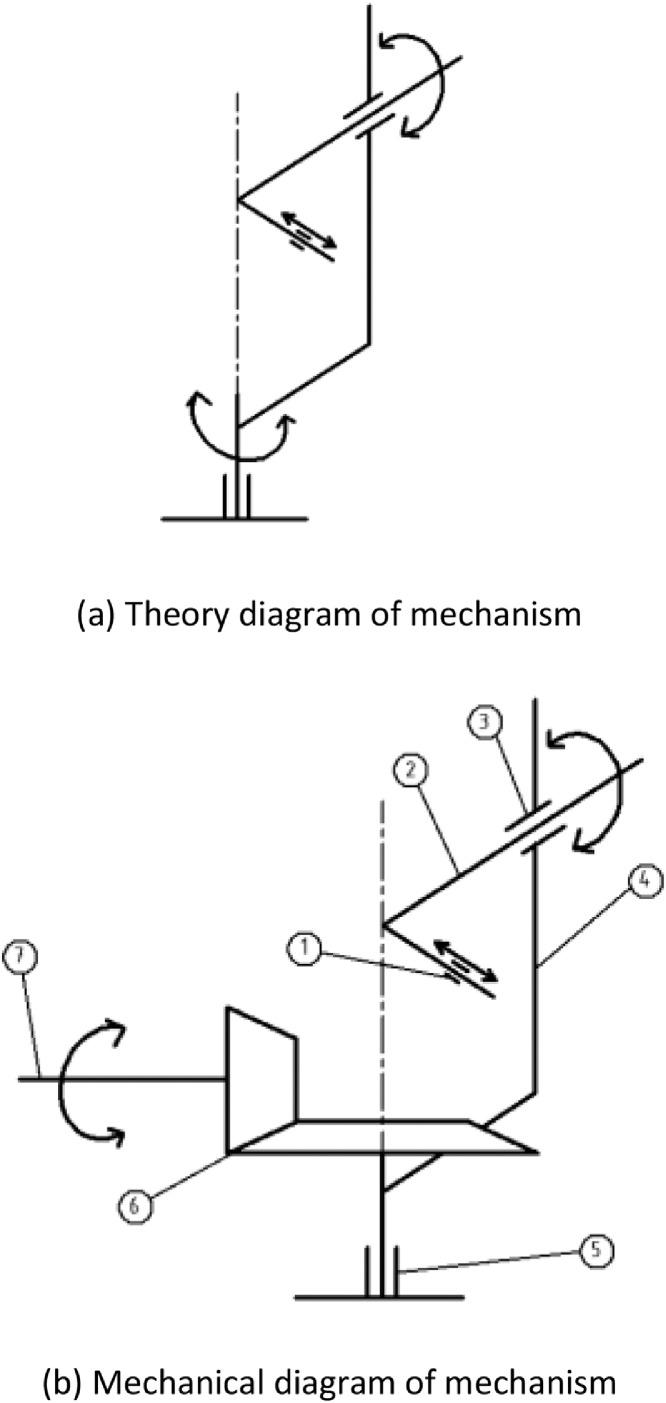
The conceptual design of proposed framework. item 1. part of linear movement from palm to wrist item 2. part of the 1st rotational DOF item 3. part of the 1st rotational joint item 4. part of mechanical body item 5. part of rotary joint item 6. part of the 2nd rotational DOF item 7. part of motor mounting mechanism.

Deriving from DOFs of hand wrist, this actuator needs to have two rotational movement to match with: one DOF for X axial rotation and the other for Z axis. After surveying the medium size of human, the dimensions of each component are proper and comfortable for each patient. During the computational process, it is acknowledged that there is essential to adjust the distance from palm to wrist. Hence, a passive DOF is attached to allow this adjustment. The novel concept of mechanical design for proposed instrument is demonstrated as Fig. 1.

The number of DOFs W in this mechanism is identified as below

(1) $$W=W_{0}-R$$

where, R: the number of constraints for dynamic joints in mechanism

$W_{0}$: the total DOFs for dynamic joints if it is separated

We have,

(2) W=6*(4−1)−4*4=2

**Fig. 2:**
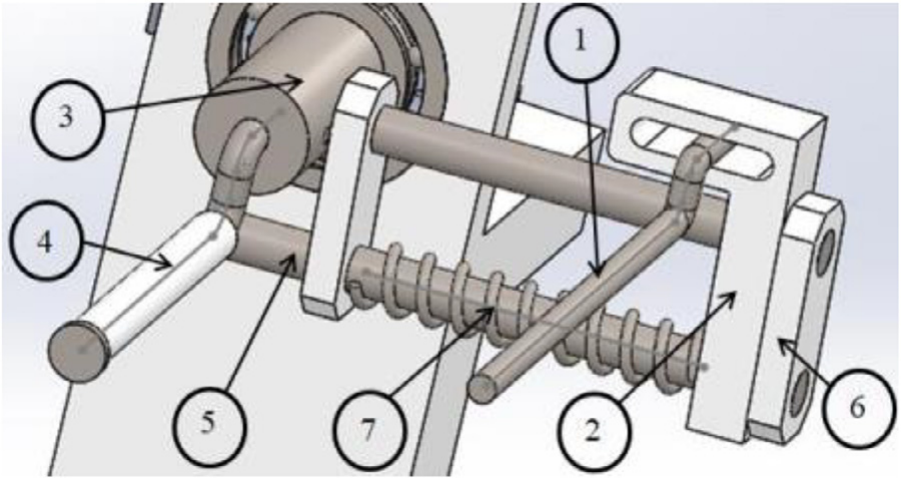
The conceptual design of proposed framework. item 1. part of holder item 2. part of fix base item 3. part of shaft connector item 4. part of rotary guide item 5. part of driving shaft item 6. part of fix beam item 7. part of lead-screw axis.

From the proposed design, mechanical model of this instrument in 3D is established as Fig. 2. Each part must obey the conditional constraints so that the total tolerances in manufacturing or assembly could be not violated.

## Computational kinetic and dynamic of proposed platform

**Fig. 3:**
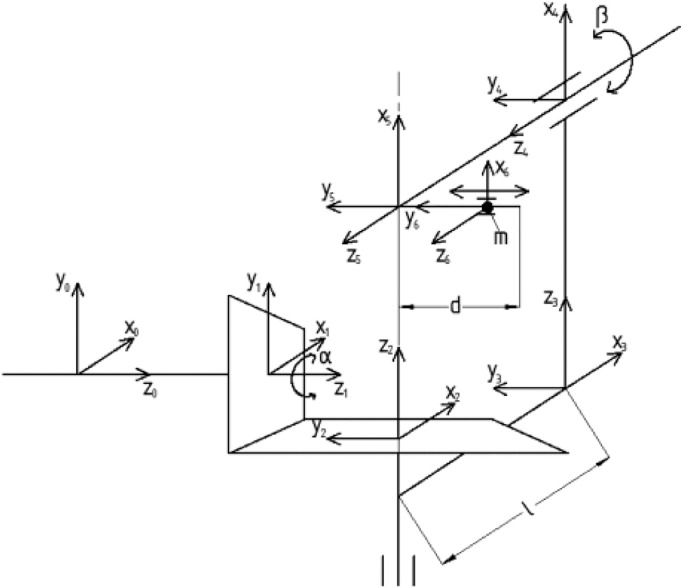
Coordinate analysis on each mechanism.

From the theoretical design, the coordinates as Fig. 3 are attached to each joint in order to compute both kinetic and dynamic for proposed instrument. Using the theory of Denavit-Hartenberg matrix, each joint has its own parameters to depict the motion as

(3) $${}_{i}^{i+1}T=\left[\begin{array}{cccc} \cos\alpha & -\sin\alpha & 0 & 0\\ \sin\alpha & \cos\alpha & 0 & 0\\ 0 & 0 & 1 & d_{i}\\ 0 & 0 & 0 & 1 \end{array}\right]$$

where, ${}_{i}^{i+1}T$: transformation matrix from link i to link i+1.

α: rotational angle around

$d_{i}$: linear slide along z axis

The transformation matrices from link 1 to link 6 are

(3) $$\begin{array}{ccc} {}_{2}^{1}T & = & \left[\begin{array}{cccc} 1 & 0 & 0 & 0\\ 0 & \cos\left(-90\right) & -\sin\left(-90\right) & 0\\ 0 & \sin\left(-90\right) & \cos\left(-90\right) & 0\\ 0 & 0 & 0 & 1 \end{array}\right]\\ & = & \left[\begin{array}{cccc} 1 & 0 & 0 & 0\\ 0 & 0 & 1 & 0\\ 0 & -1 & 0 & 0\\ 0 & 0 & 0 & 1 \end{array}\right] \end{array}$$

(4) $${}_{3}^{2}T=\left[\begin{array}{cccc} 1 & 0 & 0 & \ell \\ 0 & 1 & 0 & 0\\ 0 & 0 & 1 & 0\\ 0 & 0 & 0 & 1 \end{array}\right]$$

(5) $$\begin{array}{ccc} {}_{4}^{3}T & = & \left[\begin{array}{cccc} \cos\left(-90\right) & 0 & -\sin\left(-90\right) & 0\\ 0 & 1 & 0 & 0\\ \sin\left(-90\right) & 0 & \cos\left(-90\right) & 0\\ 0 & 0 & 0 & 1 \end{array}\right]\\ & = & \left[\begin{array}{cccc} 0 & 0 & 1 & 0\\ 0 & 1 & 0 & 0\\ -1 & 0 & 0 & 0\\ 0 & 0 & 0 & 1 \end{array}\right] \end{array}$$

(6) $${}_{5}^{4}T=\left[\begin{array}{cccc} \cos\beta & -\sin\beta & 0 & 0\\ \sin\beta & \cos\beta & 0 & 0\\ 0 & 0 & 1 & \ell \\ 0 & 0 & 0 & 1 \end{array}\right]$$

(7) $${}_{6}^{5}T=\left[\begin{array}{cccc} 1 & 0 & 0 & 0\\ 0 & 1 & 0 & -d\\ 0 & 0 & 1 & 0\\ 0 & 0 & 0 & 1 \end{array}\right]$$

Therefore, the total transformation matrix from link 1 to link 6

(8) $$\begin{array}{c} {}_{5}^{1}T={}_{2}^{1}T*{}_{3}^{2}T*{}_{4}^{3}T*{}_{5}^{4}T\\ =\left[\begin{array}{cccc} \cos\beta *sin\alpha & -\sin\alpha *sin\beta & -\cos\alpha & -d\sin\alpha *\sin\beta \\ -\cos\alpha *\cos\beta & \cos\alpha *\sin\beta & -\sin\alpha & d\cos\alpha *\sin\beta \\ -\sin\beta & \cos\beta & 1 & d\cos\beta \\ 0 & 0 & 0 & 1 \end{array}\right] \end{array}$$

**Fig. 4:**
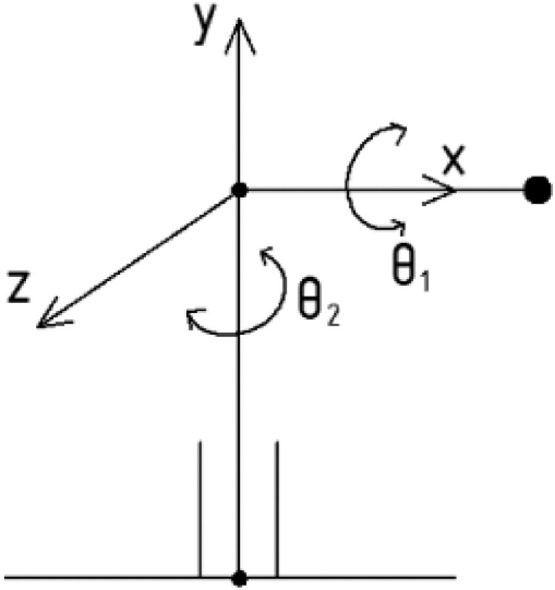
A modified diagram of theoretical design.

To simplify the dynamic computation, a theoretical diagram of proposed instrument in Fig. 4 is modified as below

The kinetic energy for rotary angle $\theta_{1},\theta_{2}$ is determined as

(9) $$K_{1}=\frac{1}{2}m{\left(l{\dot{\theta }}_{1}\right)}^{2}$$

(10) $$K_{2}=\frac{1}{2}m{\left(l{\dot{\theta }}_{2}\right)}^{2}$$

Similarly, the potential energy is

(11) $$P_{1}=mgl\sin\theta_{1}$$

(12) $$P_{2}=0$$

Using the Lagrange approach, we estimate

(13) $$\begin{array}{c} L_{m}=K-P=\left(K_{1}+K_{2}\right)-\left(P_{1}+P_{2}\right)\\ =\frac{1}{2}ml^{2}\left({\dot{\theta }}_{1}^{2}+{\dot{\theta }}_{2}^{2}\right)-mgl\sin\theta_{1} \end{array}$$

Then, the applied torques $F_{1},F_{2}$ to drive two axes are

(14) $$\left\{ \begin{array}{c} F_{1}=ml^{2}{\ddot{\theta }}_{1}+mgl\cos\theta_{1}\\ F_{2}=ml^{2}{\ddot{\theta }}_{2} \end{array}\right.$$

**Fig. 5:**
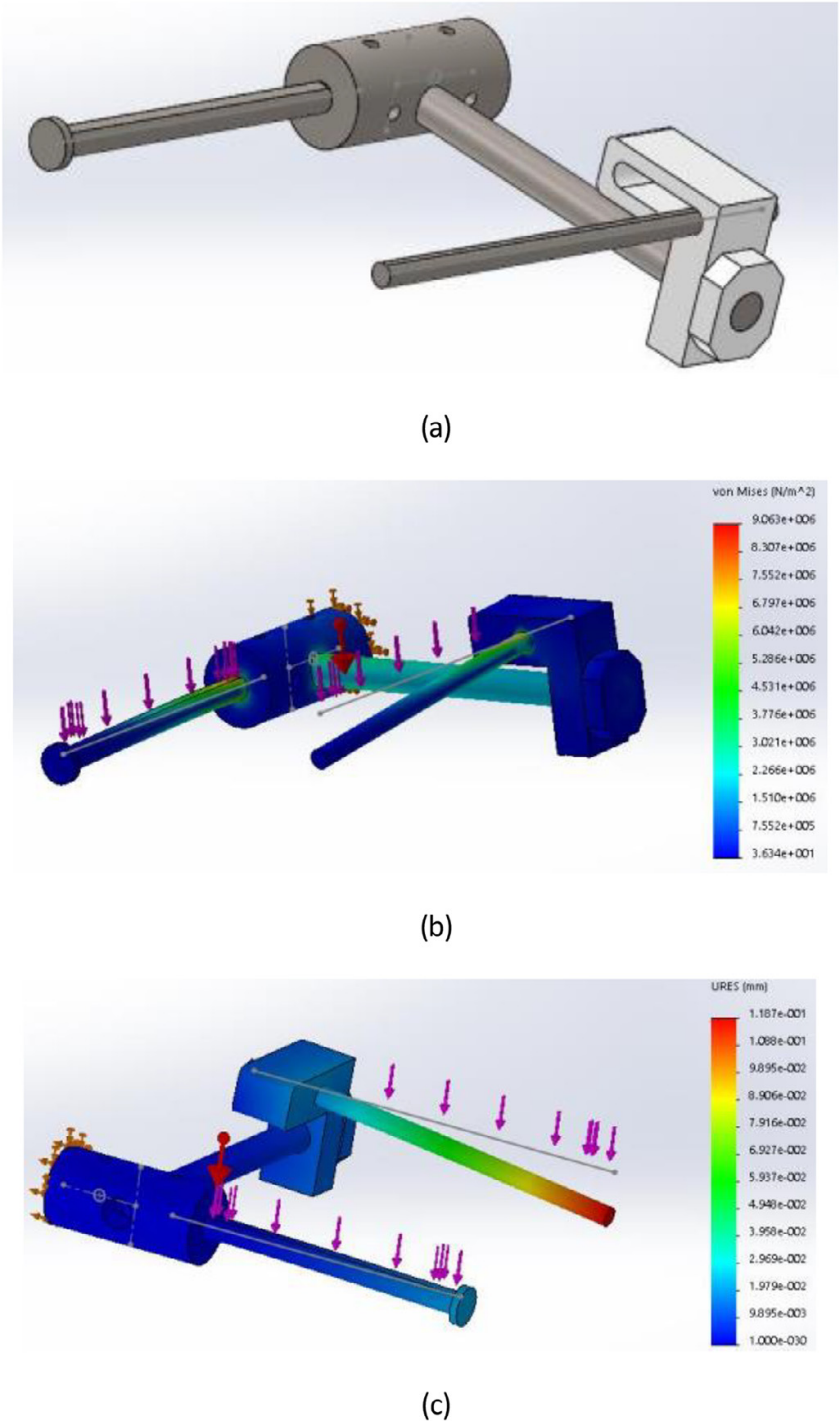
An analysis of proposed mechanism, (a) 3D model, (b) stress model and (c) transposition model.

**Fig. 6:**
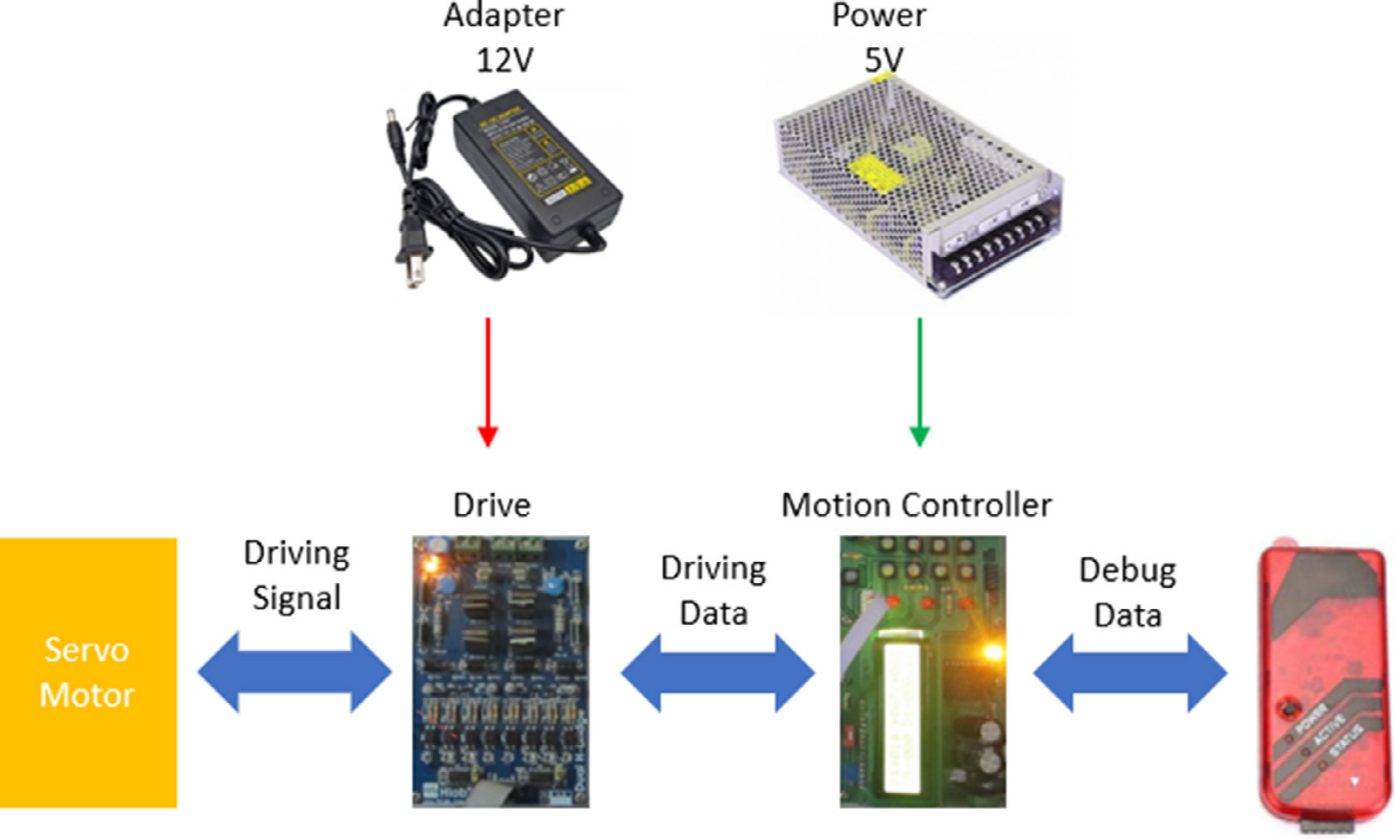
An overview of hardware design for proposed instrument.

By adopting above computation, the test validation of mechanical parts by SolidWorks software is deployed to confirm the design. The input parameters consist of materials, physical dimensions and the estimated moment at each joint. These results are shown as Fig. 5 in order to identify the maximum stress and transposition which must be smaller than thresholds (Fig. 6).

## Implementation of control scheme

**Fig. 7:**
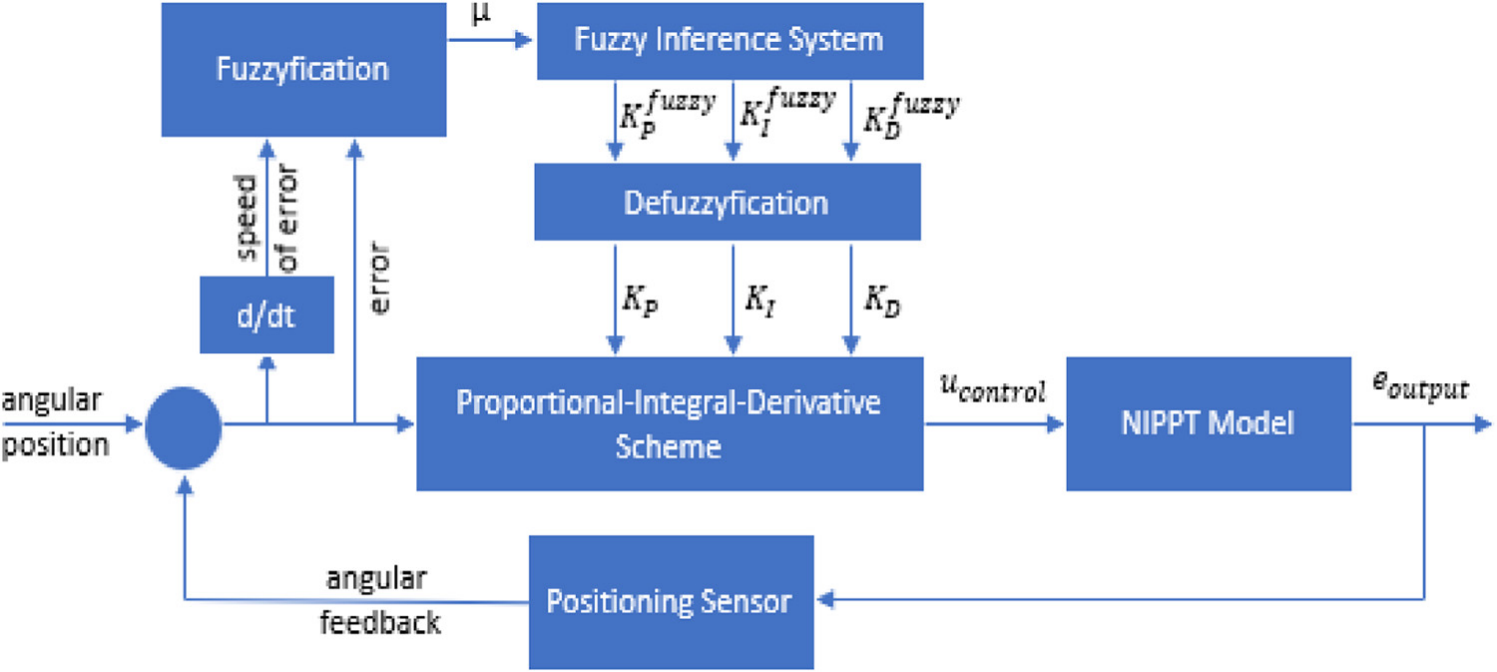
Block diagram of control scheme for proposed instrument.

Generally, the medical machine for physical therapy should have two driving motors at least. For a specific exercise, the actuator needs the sensing data, for instance torque, angular rotation and velocity, and time period. In the field of clinical knowledge, nurse or technician must determine how long the exercise is. Depending on the feedback signals from patient, sensing module could measure exactly and immediately. Nevertheless, these parameters still have the upper thresholds and lower thresholds according to each exercise, level of injury and load. As a result, the control scheme must adapt with various conditions. The fuzzy PID is initally proposed to implement in this hardware platform so that the response of driving actuator is smooth and stable. The overall scheme of proposed controller is illustrated as Fig. 7. Since this algorithm does not need the precise information of model, the signal of control output is modified frequently in respect to the current status of environment. With the F-PID control, the reasoning mechanism of control rules allows to launch the proper signals. Besides, the conventional PID control is not usually well-adapted for nonlinear model with unknown parameter variations.

The main control scheme is organized as cascade diagram such the fuzzy logic scheme is first, and PID scheme is second. The inputs of fuzzy controller contains two physical variables, error of position and derivative of error. In this case, the control requirements are very sensitive so that each adjustment could not suddenly occur.

**Fig. 8:**
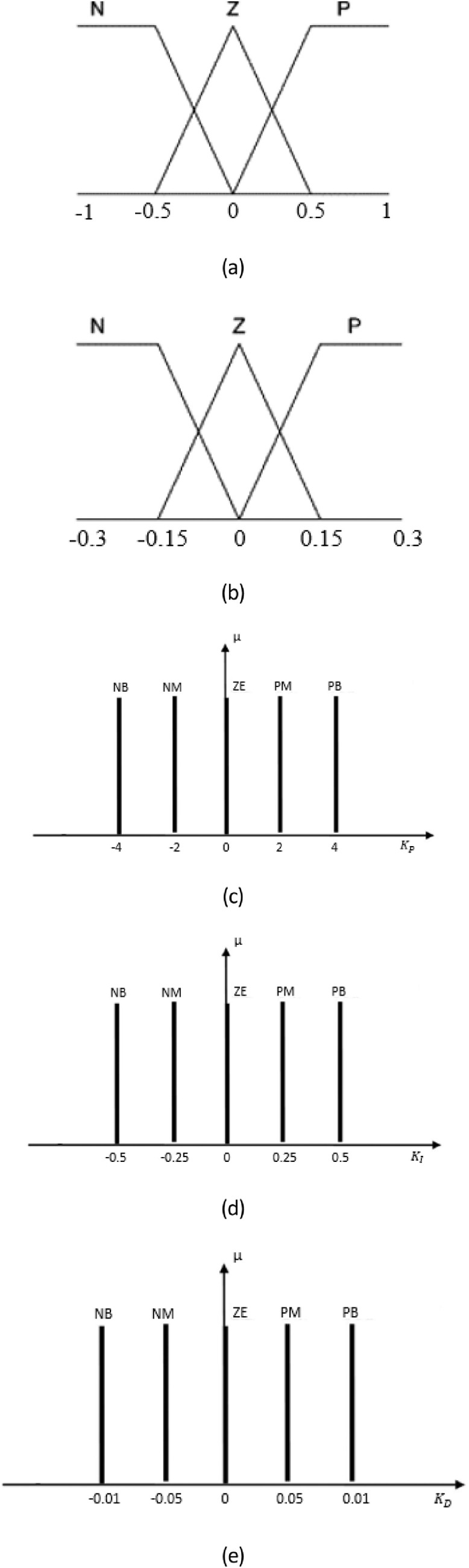
Membership functions of both inputs and outputs.

Firstly, the design process of fuzzy inference is to create the linguistic fuzzy variables as Fig. 8. The inputs of fuzzy system are error e and deviation of error $\frac{\partial e}{\partial t}$ while the outputs are fed to regulate the PID controller. The shapes of membership functions in input variables are triangle and trapezoidal because of simple and effective performance. In this design, the linguistic input variables $\begin{array}{cc} \Gamma =\{S_{i},S_{j}\} & i,j=1...3 \end{array}$ are set as negative (N), zero (Z) and positive (P). Then, $\mu_{S_{i}}\left(e\right)$ and $\mu_{S_{j}}\left(\frac{\partial e}{\partial t}\right)$ i,j=1...3 represent the corresponding membership functions. However, owing to the fast response of patient, the linguistic levels of output variables are chosen as negative big (NB), negative medium (NM), zero (ZE), positive medium (PM) and positive big (PB). Later, the membership functions for outputs $\mu_{M_{i}}$ i=1...5 have the shape of singleton in order to ensure the real-time computation. The reasoning rule-based system ℜ insists on two amtecedemts U,V and one consequence Z that are described as

$${ℜ}_{a,b,c}\;\text{IF}\;e\;\text{is}\;U_{a}\;\text{AND}\;\frac{\partial e}{\partial t}\;\text{is}\;V_{b}\;\text{then}\;\mu_{M_{i}}\;\text{is}\;Z_{c}$$

1≤a,b,c≤5

In Table 1, the fuzzy rules are recognized so that the driving command does not fluctuate unexpectedly but still adapt rapidly. For these reasons, there are totally 9 fuzzy rules to indicate each potential combination of the two fuzzy inputs to perform an output for each rule. The fuzzy inference mechanism employed the appropriate expert-knowledge to produce the crisp output $Crisp_{output}^{fuzzy}$ by fuzzification process.

(14) $$Crisp_{output}^{fuzzy}=\frac{\sum_{m=1}^{9}\mu_{M_{i}}\min\left(\mu_{S_{i}}^{m},\mu_{S_{j}}^{m}\right)}{\sum_{m=1}^{9}\min\left(\mu_{S_{i}}^{m},\mu_{S_{j}}^{m}\right)}$$

**Table 1:** Fuzzy rule-based system for evaluation.

In fact, there are some undesired phenomenons that might occur when programming in hardware level. To avoid these troubles, the upper thresholds and lower thersholds of controller outputs should be defined in order to alleviate. It is considered that $K_{P}^{upper},K_{I}^{upper},K_{D}^{upper}$ are maximum gains of PID controller respectively while $K_{P}^{lower},K_{I}^{lower},K_{D}^{lower}$ are minimum values. As a result, the actual gains are tuned as below

(15) $$K_{tuning}^{l}=\frac{K_{fuzzy}^{l}-K_{\min}^{l}}{K_{\max}^{l}-K_{\min}^{l}}$$

## Method validation

**Fig. 9:**
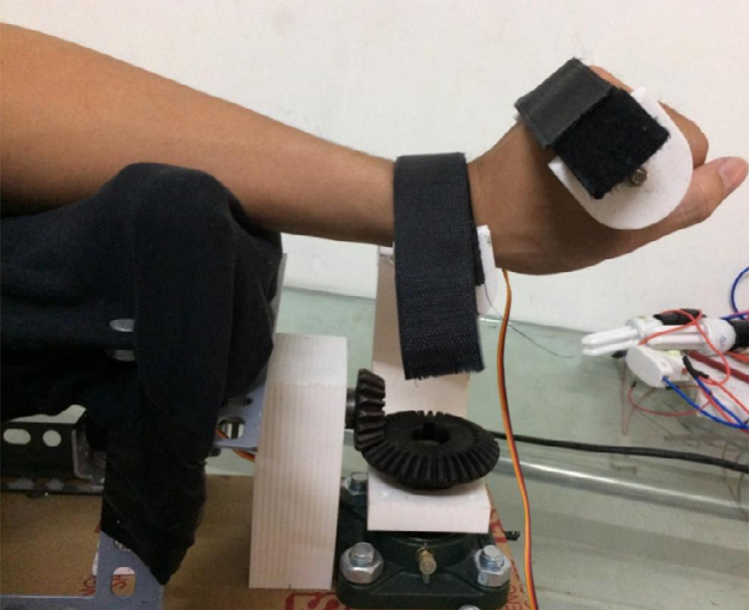
Experimental verification on different test scenarios.

In order to estimate the system performance of proposed instrument, several experiments have been conducted on real hardware as Fig. 9. One young volunteer has tried to employ this platform due to the user guideline. After the power is on, the host computer begins to communicate with main board of machine. Normally, the feedback signals including the sensing data and system state, are transmitted in one sampling period. If an operator or technician decides to switch the operating mode, then a command is passed to proposed instrument. In most cases, the security verification bit is firstly checked in the communication frame because the safety in execution is prior.

**Fig. 10:**
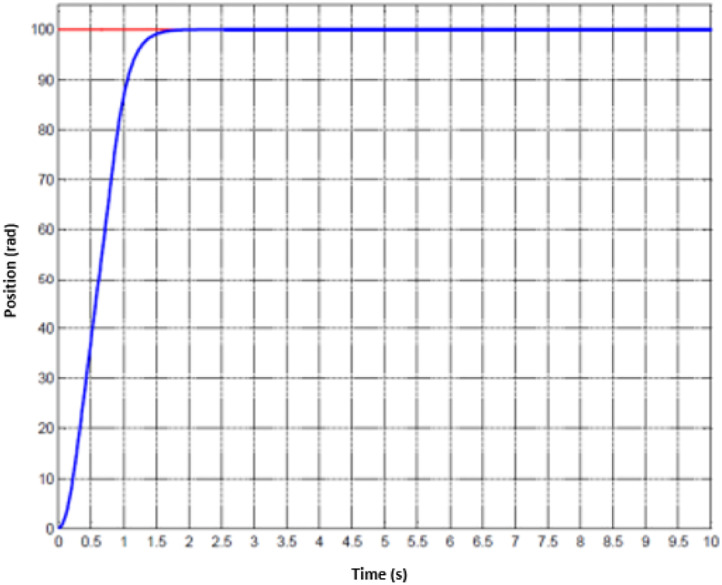
Tracking result of position performance in the first joint (red: desired value, green: actual value).

**Fig. 11:**
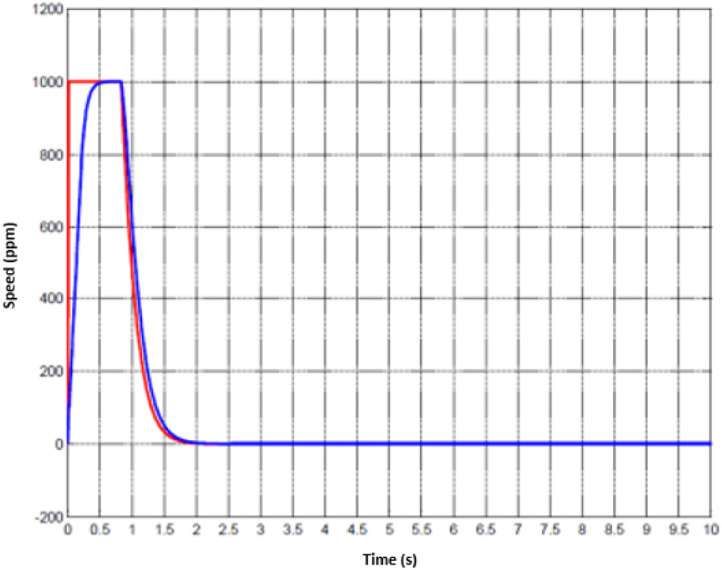
Tracking result of speed performance in the first joint (red: desired value, green: actual value).

**Fig. 12:**
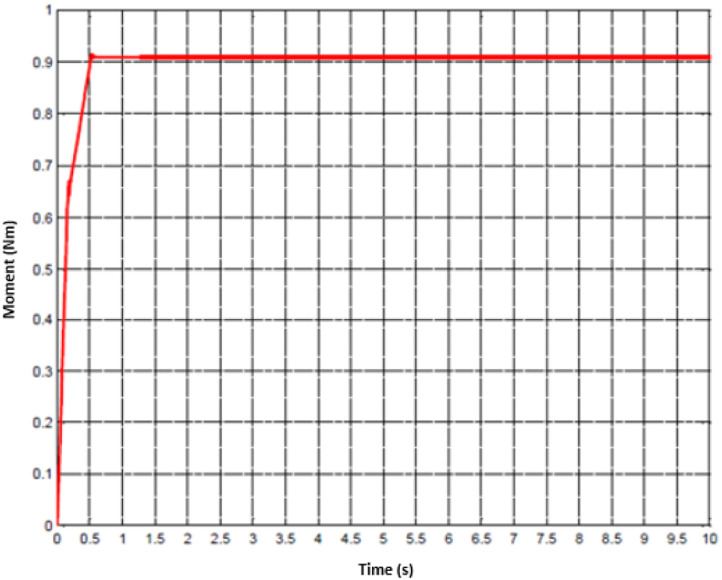
Experimental result of torque performance in the first joint.

**Fig. 13:**
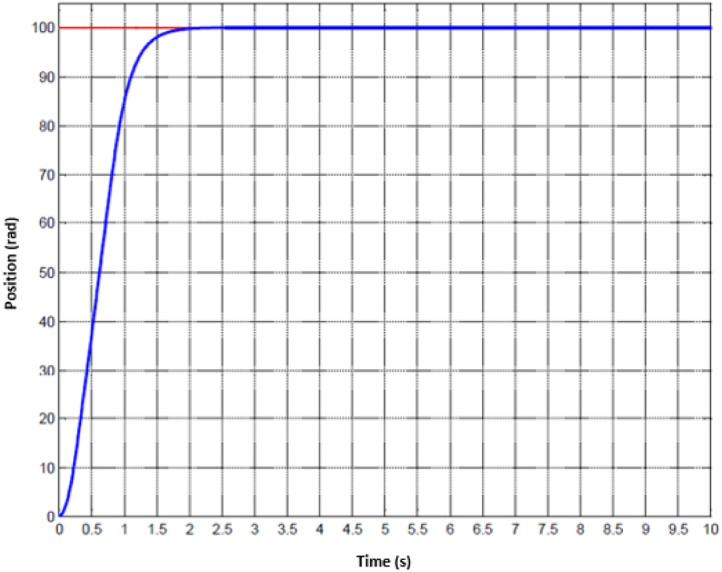
Tracking result of position performance in the second joint (red: desired value, green: actual value).

**Fig. 14:**
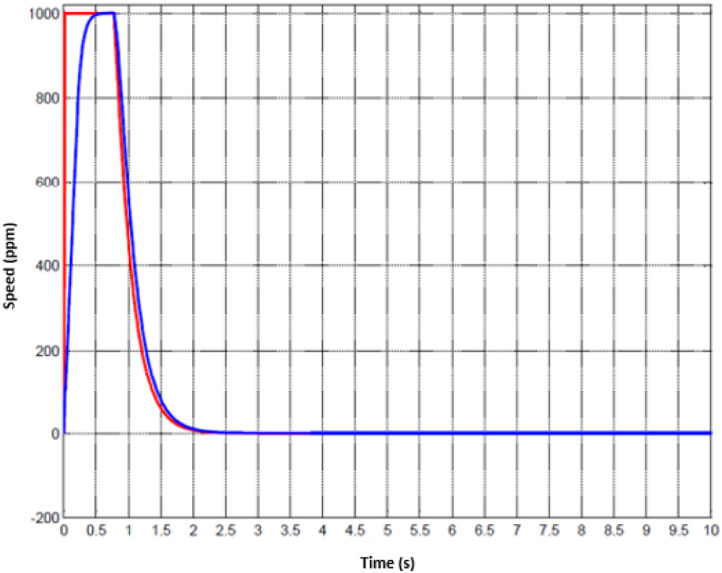
Tracking result of speed performance in the first joint (red: desired value, green: actual value).

**Fig. 15:**
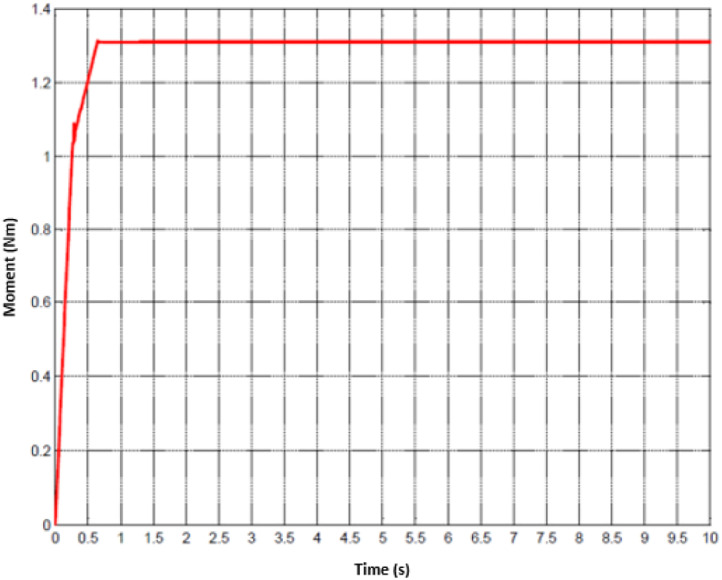
Experimental result of torque performance in the first joint.

For the treatment of injuried patient, nurse or technical operator must set the working mode in the first stage. Later, user must follow the physical exercises that are pre-determined. Whenever the movement of hand is activated, the motion parameters could be collected. Since the machine has two DOFs, hence, the status of two joints are monitored. The data of the first joint is captured from Figs. 10 to 12 whilst the others are shown from Figs. 13 to 15.

To imitate the medical therapy, the position and speed are primarily identified as the desired performance. Thereafter, the driving commands from proposed controller are fed to actuator. Thanks to F-PID scheme, the position performance in Figs. 10 and 13 for the first joint and second joint respectively, is well-received. The tracking results in Figs. 11 and 14 are also as good as the expected values. It is clearly seen that the tuning mechanism of proposed control scheme work well although the machine is under the impact of uncertainties. The torque performance of each joint is monitored in order to maintain in the range of the reasonable thresholds. From these results, the proposed design is effective, feasible and appropriate in the field of medical applications.
